# Coincident Activation of Glutamate Receptors Enhances GABA_A_ Receptor-Induced Ionic Plasticity of the Intracellular Cl^−^-Concentration in Dissociated Neuronal Cultures

**DOI:** 10.3389/fncel.2019.00497

**Published:** 2019-11-08

**Authors:** Lisa Halbhuber, Cécilia Achtner, Heiko J. Luhmann, Anne Sinning, Werner Kilb

**Affiliations:** Institute of Physiology, University Medical Center Mainz, Johannes Gutenberg University, Mainz, Germany

**Keywords:** Cl^−^-homeostasis, KCC2, reversal potential, rheobase, ionic plasticity, GABA(A) receptors, dissociated cell culture, mouse

## Abstract

Massive activation of γ-amino butyric acid A (GABA_A_) receptors during pathophysiological activity induces an increase in the intracellular Cl^−^-concentration ([Cl^−^]_i_), which is sufficient to render GABAergic responses excitatory. However, to what extent physiological levels of GABAergic activity can influence [Cl^−^]_i_ is not known. Aim of the present study is to reveal whether moderate activation of GABA_A_ receptors mediates functionally relevant [Cl^−^]_i_ changes and whether these changes can be augmented by coincident glutamatergic activity. To address these questions, we used whole-cell patch-clamp recordings from cultured cortical neurons [at days *in vitro* (DIV) 6–22] to determine changes in the GABA reversal potential (E_GABA_) induced by short bursts of GABAergic and/or synchronized glutamatergic stimulation. These experiments revealed that pressure-application of 10 short muscimol pulses at 10 Hz induced voltage-dependent [Cl^−^]_i_ changes. Under current-clamp conditions this muscimol burst induced a [Cl^−^]_i_ increase of 3.1 ± 0.4 mM (*n* = 27), which was significantly enhanced to 4.6 ± 0.5 mM (*n* = 27) when glutamate was applied synchronously with the muscimol pulses. The muscimol-induced [Cl^−^]_i_ increase significantly attenuated the inhibitory effect of GABA, as determined by the GABAergic rheobase shift. The synchronous coapplication of glutamate pulses had no additional effect on the attenuation of GABAergic inhibition, despite the larger [Cl^−^]_i_ transients under these conditions. In summary, these results indicate that moderate GABAergic activity can induce functionally relevant [Cl^−^]_i_ transients, which were enhanced by coincident glutamate pulses. This ionic plasticity of [Cl^−^]_i_ may contribute to short-term plasticity of the GABAergic system.

## Introduction

γ-amino butyric acid (GABA), the main inhibitory neurotransmitter in the adult mammalian brain, acts *via* ionotropic GABA_A_ and metabotropic GABA_B_ receptors (Mody and Pearce, 2004; Farrant and Kaila, 2007). GABA receptors not only control the excitability in the brain, but are essential for specific neuronal processes, like regulating size of neuronal assemblies, gating propagation of activity, mediating neuronal plasticity, and controlling oscillatory activity (Whittington and Traub, 2003; Fagiolini et al., 2004; Jonas et al., 2004; Mody and Pearce, 2004; Pouille and Scanziani, 2004). As ligand-gated chloride channels, GABA_A_ receptors permit in the adult nervous system Cl^−^ influx, which hyperpolarizes the membrane and mediates an inhibitory action. In addition, the opening of GABA_A_ receptors induces shunting inhibition due to a decreased membrane resistance (Farrant and Kaila, 2007). The Cl^−^ influx, and thus the inhibitory hyperpolarization of the membrane potential, depends on a negative equilibrium potential for Cl^−^ (E_Cl_), which is determined by a low intracellular chloride concentration ([Cl^−^]_i_). This low [Cl^−^]_i_ is maintained by the chloride extruder potassium chloride cotransporter 2 (KCC2) in the adult mammalian brain (Rivera et al., 1999, 2005; Blaesse et al., 2006, 2009). In accordance with the central role of KCC2 for the function of the GABAergic systems, dysfunctions of Cl^−^ extrusion has been linked to neurological diseases, like epilepsy or chronic pain (Kahle et al., 2008; Kaila et al., 2014a; Silayeva et al., 2015). Thus, KCC2 has been identified as a putative target for anticonvulsive therapies (Löscher et al., 2013; Puskarjov et al., 2014a; Moore et al., 2018) and pain control (Gagnon et al., 2013; Kahle et al., 2014a; Lavertu et al., 2014).

As GABA_A_ receptors mediate a considerable Cl^−^ conductance, they directly affect [Cl^−^]_i_, a process that is termed "ionic plasticity" (Rivera et al., 2005; Jedlicka and Backus, 2006; Wright et al., 2011; Raimondo et al., 2012b; Kaila et al., 2014a). It has been shown that massive GABAergic activity induces considerable and functionally relevant changes in [Cl^−^]_i_ (Ballanyi and Grafe, 1985; Thompson and Gähwiler, 1989; Kuner and Augustine, 2000; Fujiwara-Tsukamoto et al., 2003; Isomura et al., 2003; Raimondo et al., 2015; Moore et al., 2018). In the adult CNS massive GABAergic activity led to a [Cl^−^]_i_ increase, which depends on HCO_3_^−^ gradients and additional [K^+^]_e_ transients (Staley et al., 1995; Kaila et al., 1997). However, there is little evidence that also moderate levels of GABAergic activity can mediate functionally relevant [Cl^−^]_i_ changes in the mature nervous system (Kaila et al., 1997). In contrast, already physiological levels of GABAergic activity affect [Cl^−^]_i_ the immature nervous system (Kolbaev et al., 2011b; Lombardi et al., 2018), in which the steady-state [Cl^−^]_i_ is high (Cherubini et al., 1991; Ben-Ari, 2002). These transient [Cl^−^]_i_ changes after limited GABAergic stimulation is most probably due to the low capacity of NKCC1-mediated Cl^−^ accumulation in these neurons (Achilles et al., 2007). The activity-dependent [Cl^−^]_i_-decrease in the immature nervous system serves to limit the excitatory action of GABA (Ben-Ari et al., 2012; Kilb et al., 2013). But in the adult situation, the activity-dependent [Cl^−^]_i_ increase attenuates the inhibitory potential of GABA and, in case of a strong GABAergic activity, could even render GABA excitatory (Staley et al., 1995; Kaila et al., 2014b).

The activity-dependent [Cl^−^]_i_ changes depend on the activity of [Cl^−^]_i_ transport mechanisms, the conductance and distribution of Cl^−^ channels, the diameter and topology of dendrites, as well as on distance of synaptic sites from the soma (Doyon et al., 2011; Jedlicka et al., 2011; Kaila et al., 2014a; Mohapatra et al., 2016; Lombardi et al., 2019). Thus, activity-dependent [Cl^−^]_i_ changes are mainly restricted to the dendritic compartment (Doyon et al., 2011; Jedlicka et al., 2011). The amount of Cl^−^ fluxes during a GABAergic event is determined by the difference between E_m_ and E_Cl_ [i.e., the driving force for Cl^−^ (DF_Cl_; Ballanyi and Grafe, 1985; Backus et al., 1998; Kuner and Augustine, 2000; Jedlicka and Backus, 2006]. Accordingly, stabilization of E_m_ by a low input resistance (Lombardi et al., 2019) or a positive shift in E_GABA_ due to the contribution of HCO_3_^−^ to GABAergic currents (Rivera et al., 2005; Wright et al., 2011; Lombardi et al., 2019) enhances the activity-dependent [Cl^−^]_i_ changes in neurons. Since the synchronous stimulation of glutamate receptors will depolarize E_m_ and therefore enhance DF_Cl_, a coincident activation of glutamate receptors will enhance the Cl^−^ fluxes through GABA_A_ receptors. While it has already been demonstrated that a depolarization indeed enhances GABAergic [Cl^−^]_i_ transients (Ballanyi and Grafe, 1985; Thompson and Gähwiler, 1989; Kuner and Augustine, 2000) and it is obvious that a massive glutamatergic stimulation will thus lead to larger [Cl^−^]_i_ transients (Moore et al., 2018), it has not been shown experimentally whether moderate levels of glutamatergic activity are indeed sufficient to substantially enhance GABAergic [Cl^−^]_i_ transients. In addition, it is currently unknown whether moderate levels of ionic plasticity in [Cl^−^]_i_ significantly affect GABAergic inhibition, although some studies demonstrate that massive GABAergic stimulation can render GABA excitatory *via* ionic plasticity (Staley et al., 1995; Fujiwara-Tsukamoto et al., 2003; Isomura et al., 2003).

To address the question, whether coincident activation of GABA_A_ and glutamate receptors mediate increased ionic plasticity, we induced short trains of GABA_A_ receptor activations, applied either with or without synchronous glutamatergic activation, and analyzed the resulting changes in [Cl^−^]_i_. To further investigate whether these [Cl^−^]_i_ changes lead to a functionally relevant reduction of inhibitory capacity, we additionally determined whether these stimulation paradigms change the GABAergic rheobase shift. These experiments revealed that GABAergic activity leads to a [Cl^−^]_i_ increase and attenuates the inhibitory effect of GABA. While coincident glutamate receptor activation augments the activity-dependent [Cl^−^]_i_ shifts, it does not have additional effect on the activity-induced decrease in GABAergic inhibitory capacity.

## Materials and Methods

### Preparation of Dissociated Neuronal Cultures

Experiments were performed in primary cortical neurons cultured from newborn (postnatal day 0) mice (C57BL6). After decapitation brains were transferred to ice-cold Ca^2+^- and Mg^2+^-free HBSS (Gibco, Invitrogen, Carlsbad, CA, USA) supplemented with penicillin and streptomycin (50 units/ml), sodium pyruvate (11 mg/ml), glucose (0.1%), and HEPES (10 mM). Cortical cells were dissociated *via* trypsin incubation for 20 min at 37°C and DNAse digestion at room temperature (RT). After blocking trypsinization by washing steps with HBSS, Minimal Essential Medium (MEM, Gibco) supplemented with 10% horse serum and 0.6% glucose was added. Next the cells were mechanically dissociated *via* repetitive pipetting through fire-polished glass pipettes with declining diameter. Following cell counting, cells were seeded on Polyornithine-coated glass coverslips in a 24-well-plate (density: 1,000 cells/mm^2^). After 45 min, the medium was exchanged for medium consisting of Neurobasal medium (Gibco) supplemented with 2% B27 (Gibco) and 1 mM L-glutamine.

Cells were cultivated at 37°C in humidified carbogen (95% air; 5% CO_2_) for up to 22 days. At 3 days *in vitro* (DIV) 5 μM AraC was added to the medium to inhibit glial cell proliferation. A quarter of the medium was exchanged weekly.

### rtPCR

Total RNA from primary cortical cultures was isolated using the RNeasy Mini Kit (Qiagen). mRNA was reverse transcribed using the Transcriptor High Fidelity cDNA Synthesis Kit (Roche Applied Science). qRT-PCR was performed using the LightCycler TaqMan Master Kits as well as probes and primers designed with the Universal Probe Library in a LightCycler 1.5 System (all Roche Applied Science). Primer sequences (in 5′–3′orientation) of target gene and probes are as follows: KCC2 (UPL probe #79), TTCGACCCACCCAATTTC and AAAGCCATGGCGAGACAG; β-actin (UPL probe#106), TGACAGGATGCAGAAGGAGA and CGCTCAGGAGGAGC-AATG. The qRT-PCR crossing points were used for relative quantification based on the ΔCt-method using the StepOne software (version 2.3) and β-actin was used as a reference gene (Pfaffl et al., 2002).

### Immunohistochemistry

Primary cortical neurons grown on coverslips were fixed in 4% PFA in PBS for 20 min after 7 or 14 days in culture. After washing with PBS unspecific binding of antibodies was blocked with 7% normal donkey serum and 0.3% Triton diluted in PBS for 2 h (RT). Overnight staining was performed at 4°C with rabbit anti-KCC2 antibody (Sigma Aldrich, #07-432) diluted 1:500 in 2% bovine serum albumin with 0.05% azide and 0.1% Triton. After subsequent wash with PBS, cells were incubated with DAPI and DyLight488-coupled secondary antibodies (Dianova and Biomol, Hamburg, Germany) diluted 1:500 in 2% bovine serum albumin with 0.05% azide in PBS at RT for 2 h. Coverslips were washed in PBS and specimens were mounted with Fluoromount (Sigma-Aldrich).

### MEA Recordings

Extracellular electrical recordings were performed as described previously (Weir et al., 2015). In short, cells were cultured on MEAs containing 120 planar extracellular titanium nitrite electrodes with four internal references (120MEA100/30iR-Ti-gr, Multi Channel Systems). MEAs had an electrode diameter of 30 μm and an interelectrode spacing of 100 or 200 μm. Signals from 120 recording electrodes were recorded with MC_Rack software in a MEA 2100 system (Multi Channel Systems) at a sampling rate of 50 kHz and high-pass filtered at 200 Hz. Spikes were detected using a negative threshold-based detector set to a threshold of 7× the SD of the noise level (MC_Rack, Multi Channel Systems). Electrophysiological recordings were performed in artificial cerebrospinal fluid (ASCF) resembling the medium composition consisting of (in mM) 129 NaCl, 26 NaHCO_3_, 1 MgCl_2_, 2 CaCl_2_, 5.3 KCl, 10 glucose (equilibrated with 95% O_2_/5% CO_2_). Temperature was maintained at 32°C by a temperature controller (TC02, Multi Channel Systems). Spike datasets from all electrodes recorded for 10 min were imported into Matlab 7.7 (Mathworks, Natick, MA, USA) for analysis of single units using a custom written routine. Spike sorting was carried out as described previously (Sun et al., 2010). Autocorrelation functions were applied to confirm spike sorting. If single units fired ≥2 times within the recording period, units were counted as active neurons. Average firing frequencies were calculated as arithmetic mean of individual firing frequencies of all identified units.

### Whole-Cell Patch-Clamp Recording

For patch-clamp experiments, cultured cells were transferred into ACSF that consisted of (in mM) 126 NaCl, 26 NaHCO_3_, 1.25 NaH_2_PO_4_, 1 MgCl_2_, 2 CaCl_2_, 2.5 KCl, 10 glucose and was equilibrated with 95% O_2_/5% CO_2_ (pH = 7.4; osmolarity = 316 mOsm). In the experimental setup, cells were constantly perfused with ACSF at a rate of 2 ml/min. All experiments were performed at 31 ± 1°C.

Cell cultures were visualized using an inverted microscope (BX51WI, Olympus) equipped with a CCD camera (VX45, Optronics, Goleta, CA, USA) connected to a video monitor (PVM-145E, Sony). Pictures of each cell were taken to be able to reconstruct morphology and pipette positioning. Only cells with clearly visible dendritic extensions downstream the bath perfusion and facing the insertion site of the application pipettes were chosen to minimize steric problems while positioning the pipettes. The patch electrode, as well as one of the application pipettes, were positioned using electric manipulators (SM-1, Luigs and Neumann, Ratingen, Germany), while the second application pipette was mounted on manual manipulators. The application pipettes were positioned in the dendritic tree ~100 μm apart from the soma downstream the bath perfusion to make sure that no other cell compartments are superfused by the applied substances *via* the perfusion system. Muscimol (200 μM, 10 ms) and glutamate pulses (200 μM, 20 ms) were delivered by separate pipettes, while muscimol application for E_GABA_ determination and burst like GABA_A_ receptor activation was delivered *via* the same pipette. Focal application was conducted *via* a custom-built pressure application system (Lee, Westbrook, CT, USA) at a pressure of 0.5 bar. To impede the removal of focally applied neurotransmitter, an additional suction pipette was placed close to the application sites by a manual manipulator.

Patch-clamp recordings were conducted with a discontinuous voltage clamp/current clamp amplifier (SEC05L, NPI, Tamm, Germany), connected to a standard personal computer *via* a digital-analog converter (LIH1600, HEKA Electronics, Lambrecht, Germany). Both, recording data for later analysis and executing protocols were mediated by TIDA software (TIDA 5.25, HEKA). Patch electrodes and application pipettes were pulled from borosilicate glass capillaries (GB200F-8, Science Products, Hofheim am Taunus, Germany) using a vertical electrode puller (Model PP-830, Narishige Co., Tokyo, Japan). For the majority of whole-cell recordings, the patch electrodes (resistance 3–5 MΩ) were filled with a K-gluconate based pipette solution containing 10 mM Cl^−^ (128 K-gluconate, 4 KCl, 1 CaCl_2_, 4 NaCl, 11 EGTA, 10 K-HEPES, pH adjusted to 7.4 with KOH and osmolarity to 300 mOsm with sucrose). Few control experiments were performed with a high Cl^−^ pipette solution containing 20 mM Cl^−^ (118 K-gluconate, 14 KCl, 1 CaCl_2_, 4 NaCl, 11 EGTA, 10 K-HEPES, pH adjusted to 7.4 with KOH and osmolarity to 300 mOsm with sucrose) or 50 mM Cl^−^ (88 K-gluconate, 44 KCl, 1 CaCl_2_, 4 NaCl, 11 EGTA, 10 K-HEPES, pH adjusted to 7.4 with KOH and osmolarity to 300 mOsm with sucrose). All potentials were corrected for liquid junction potential of −14.4 mV for 10 mM Cl^−^ solution, −13.5 for 20 mM Cl^−^ solution and −10.7 mV for 50 mM Cl^−^ solution.

To estimate [Cl^−^]_i_, the GABA reversal potential was determined by either muscimol applications (200 μM, 10 ms) at different holding potentials (−80, −60 and −40 mV) or by paired voltage ramps (−80 to −20 mV; 50 ms; 2 V/s), one applied at control conditions and a second during the peak of a muscimol response, induced by a single application of muscimol (Figures 2A,B). To minimize the influence of these muscimol induced currents on [Cl^−^]_i_, we only used three muscimol pulses at holding potentials between −80 and −40 mV. E_GABA_ was estimated either from the intersection of the linear fit with the abscissa for voltage step determination and from the intersection of the control voltage ramp response and the voltage ramp response during muscimol peak (Figure 2C). [Cl^−^]_i_ was calculated from E_GABA_ using the Nernst equation. The experiments with voltage-ramp E_GABA_ determination were performed with 0.1 μM TTX to prevent AP generation by the voltage ramps and reduce spontaneous activity. Spontaneous excitatory (EPSCs) and inhibitory postsynaptic currents (IPSCs) were recorded with a 10 mM [Cl^−^]_p_, were isolated by their reversal potential, and were analyzed with Minianalysis software (Synaptosoft, Fort Lee, NY, USA).

**Figure 1:**
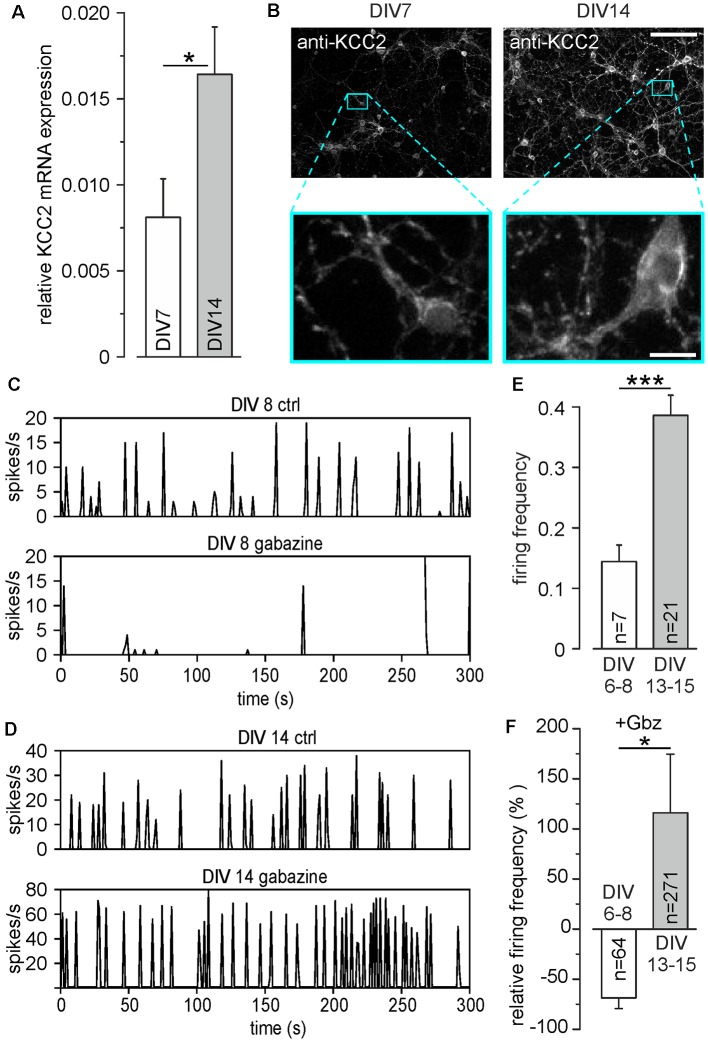
Development of inhibitory γ-amino butyric acid (GABA) responses in dissociated cell cultures during the first 15 DIV. **(A)** Relative mRNA levels of potassium chloride cotransporter 2 (KCC2) at different days *in vitro* (DIV). **(B)** Antibody staining against KCC2 at DIV 7 and DIV14. The lower panels show a 10× magnification of the marked areas in the upper panels. Note the strong increase of KCC2 expression, especially in dendritic compartments. Scale bars: 100 μm (upper panels); 10 μm (lower panels). **(C)** Representative MEA recordings at DIV 8 under control conditions and in the presence of 20 μM gabazine. **(D)** Representative MEA recordings at DIV 14 under control conditions and in the presence of 20 μM gabazine. **(E)** Average firing frequency of all units recorded on MEAs, measured at DIV 6–8 and DIV 13–15, respectively. Note that compared to DIV 6–8, the average firing frequency was significantly increased at DIV 13–15. **(F)** Effect of the GABA receptor antagonist gabazine (Gbz, 20 μM) on firing frequency. Gabazine decreased the firing frequency at DIV 6–8, while it led to an increase at DIV 13–15. Bars represent mean ± SEM, *,***indicate significance levels of *p* < 0.05 and *p* < 0.001, respectively.

**Figure 2:**
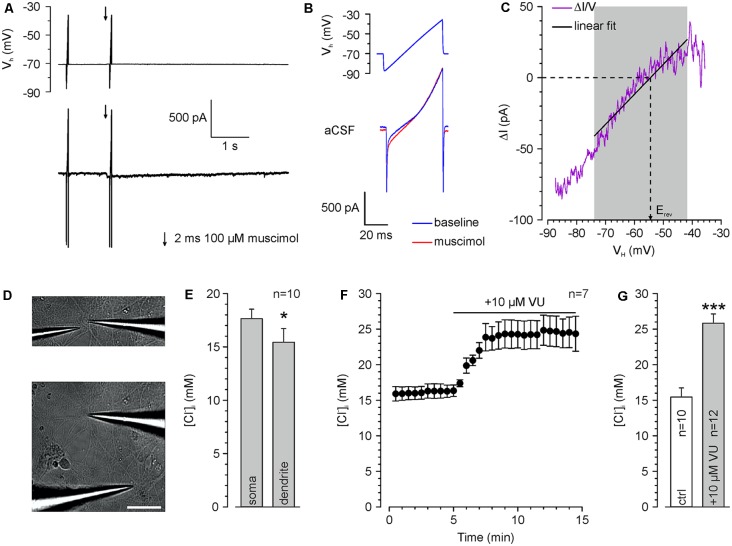
Determination of somatic and dendritic [Cl^−^]_i_ in whole-cell patch-clamp experiments. **(A)** Typical current traces upon injection of voltage ramps which are used to determine E_Cl_. The second voltage ramp was applied during the peak of a muscimol response. **(B)** Voltage ramp and current response is shown in **(A)** at higher temporal resolution. Note the divergent current amplitude in the presence of muscimol (red trance). **(C)** Relation between muscimol-dependent current difference (ΔI = I_Muscimol_ − I_Baseline_) and the holding potential (V_H_). E_rev_ was determined from a linear fit between 14 and 44 ms. **(D)** Brightfield pictures of a recorded cell illustrating application pipettes positioned near the soma (upper panel) and at the dendrite (lower panel). Scale bar: 50 μm. **(E)** Resting [Cl^−^]_i_ at the soma compared to a dendritic compartment 100 μm apart from the soma center. All experiments were performed under whole-cell conditions with a pipette solution containing 20 mM Cl^−^ ([Cl^−^]_p_). Note the significant lower [Cl^−^]_i_ in the dendrite. **(F)** Time course of dendritic E_rev_ after inhibition of KCC2 with 10 μM VU 0463271. Note the strong [Cl^−^]_i_ increase upon KCC2 inhibition. **(G)** Dendritic [Cl^−^]_i_ at rest and after VU 0463271 application (≥30 min). Note that VU 0463271 induced a significant increase in [Cl^−^]_i_. Bars/data points represent mean ± SEM. *,***Indicate significance levels of *p* < 0.05 and *p* < 0.001, respectively.

**Figure 3:**
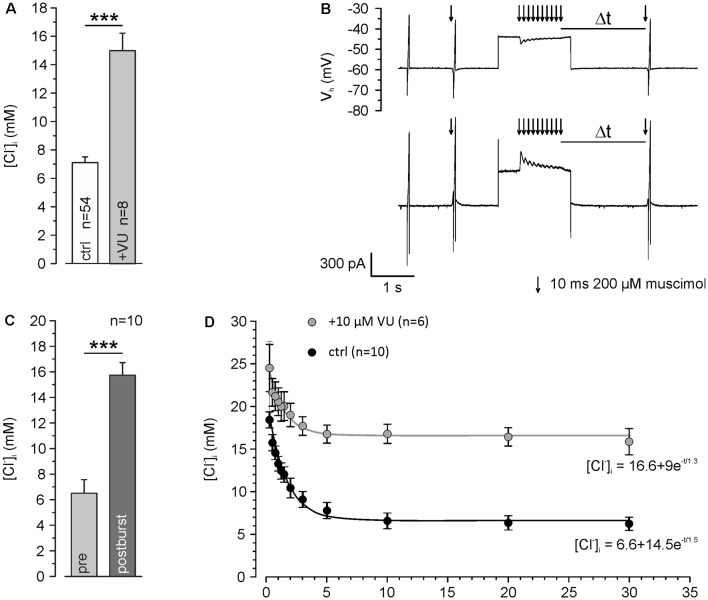
KCC2 transport activity maintains low [Cl^−^]_i_ and speeds up backregulation of GABA_A_ receptor-mediated [Cl^−^]_i_ increase. **(A)** [Cl^−^]_i_ using a [Cl^−^]_p_ of 10 mM under control conditions and ≥30 min after washing of 10 μM VU 0463271. Note that even under this condition resting [Cl^−^]_i_ was maintained below [Cl^−^]_p_ and that this effect depends on KCC2. **(B)** Original traces illustrating the stimulation paradigm. [Cl^−^]_i_ was manipulated by a 10 Hz burst of single muscimol pulses (10 ms, 200 μM) while clamping the cell at a depolarized holding potential of −40 mV. The reversal potential of GABA_A_ receptors was measured at different latencies (Δt) after the muscimol burst. **(C)** Statistical analysis revealing that [Cl^−^]_i_ was significantly increased 500 ms after the end of the burst. **(D)** [Cl^−^]_i_ determined at different Δt. Note that [Cl^−^]_i_ decreased to control values after about 5 s and that this regulation was strongly depressed in the presence of VU 0463271. Bars and data points represent mean ± SEM, data points represent mean ± SEM or single-cell values. ***Indicates significance levels of *p* < 0.001.

For “burst-like” focal application, a train of 10 single pulses at a frequency of 10 Hz was applied at a holding potential of −60 mV in current-clamp mode. In few control experiments, cells were voltage-clamped during the burst-like focal application. The slope of [Cl^−^]_i_ back regulation was determined by fitting the [Cl^−^]_i_ decay with a monoexponential function and calculating the apparent [Cl^−^]_i_ changes between the arbitrary [Cl^−^]_i_ values of 17.5 mM and 18.5 mM (Figure 3D). The rheobase [i.e., the minimal injection current required to trigger action potentials (AP)] and the muscimol-induced rheobase shift were determined using paired current ramps of 50 ms duration with 2 nA maximum current and separated by a 1 s interval and applied a muscimol pulse (200 μM, 10 ms) during the second ramp. Muscimol application was temporally aligned to the voltage ramp for each cell separately, to obtain conditions where the current ramp is delivered during the peak of the muscimol response (Kolbaev et al., 2011a). Using this protocol, the rheobase for the first (I_RB_^ctrl^) and the second (I_RB_^GABA^) current ramp was determined and the relative rheobase shift (ΔI_RB_ = I_RB_^muscimol^ − I_RB_^ctrl^) was calculated (see Figure 6A). I_RB_^ctrl^ was used to determine whether repetitive GABAergic inputs affect the excitability of the cell.

**Figure 4:**
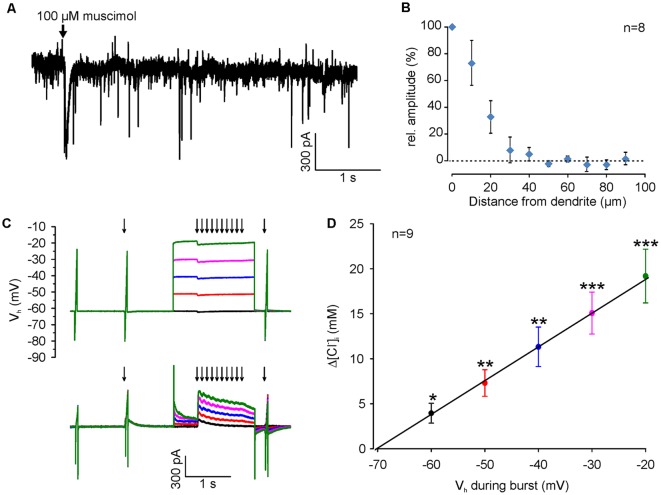
[Cl^−^]_i_ increase upon burst-like GABA_A_ receptor activation presents strong voltage-dependence. **(A)** Typical current trace of a neuron (recorded at −60 mV with a [Cl^−^]_p_ of 50 mM) illustrating spontaneous synaptic currents and the inward current induced by focal application of 100 μM muscimol (arrow). Note that the evoked response had a slightly larger amplitude but last considerably longer than spontaneous currents. **(B)** Relative amplitude of muscimol-induced currents at various distances from the dendrite. Note the absence of evoked currents at distances >40 μm. **(C)** Original traces illustrating the stimulation paradigm. [Cl^−^]_i_ was manipulated by a 10 Hz burst of single muscimol pulses (2 ms, 100 μM), while clamping the cell at different depolarized holding potentials from −60 to −20 mV. **(D)** The increase in [Cl^−^]_i_ (Δ[Cl^−^]_i_) detected 500 ms after the end of the burst is strictly voltage-dependent. Data points represent mean ± SEM. Asterisks on top of values indicate significance from 0. *,**,***Indicate significance levels of *p* < 0.05, *p* < 0.01 and *p* < 0.001, respectively.

**Figure 5:**
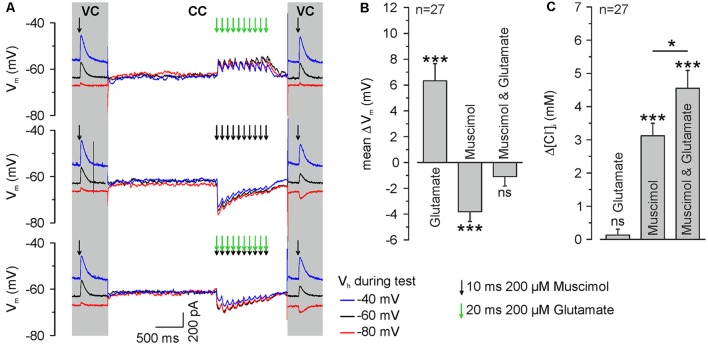
GABA_A_ receptor activation induces ionic plasticity, which is further increased by coincident glutamate receptor activation. **(A)** Original traces illustrating the stimulation paradigm. [Cl^−^]_i_ was calculated from E_Rev_ determined from single muscimol pulses applied before and after burst application at different V_H_ under voltage-clamp conditions (VC). The burst application occurred under current-clamp conditions (CC) and consisted of 10 single pulses of 200 μM muscimol and/or 200 μM glutamate provided at a frequency of 10 Hz. Muscimol and glutamate applications are marked by black or green arrows, respectively. **(B)** Statistical analysis of mean change in V_m_ (ΔV, averaged over burst duration) induced by application of 200 μM muscimol and/or 200 μM glutamate. A significant effect of muscimol and/or glutamate on the depolarization (*F*_(2,78)_ = 29.54, *p* < 0.001) was determined with a one-way ANOVA test. Note that glutamate applications induced a significant depolarization, whereas muscimol induced a hyperpolarization and the combined application did not induce a mean membrane potential change. **(C)** Statistical analysis of [Cl^−^]_i_ change after burst application of muscimol and/or glutamate. A significant effect of muscimol and/or glutamate on [Cl^−^]_i_ changes (*F*_(2,78)_ = 34.41, *p* < 0.001) was determined with a one-way ANOVA test. Note that glutamate bursts did not lead to a change in [Cl^−^]_i_, whereas muscimol and the combined muscimol/glutamate application increased [Cl^−^]_i_. Bars represent mean ± SEM. *,***Indicate significance levels of *p* < 0.05 and *p* < 0.001, respectively; ns, indicate non-significant results.

**Figure 6:**
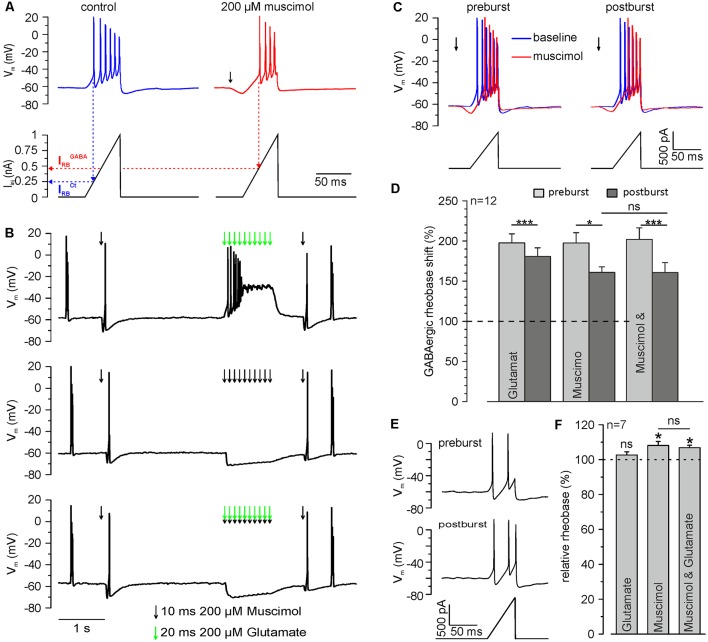
Repetitive GABA_A_ receptor activation reduces the inhibitory capacity of GABA_A_ receptors. **(A)** Original trace illustrating the determination of the rheobase by current ramp application under control conditions (blue trace) and at the peak of a single muscimol response (red trace). The rheobase (I_RB_) is defined by the injection current required to trigger the first action potential (dashed lines). Note than upon muscimol application I_RB_ increases, indicating an inhibitory effect. **(B)** Original trace illustrating the determination of the rheobase shift by current ramp application before and 600 ms after muscimol and/or glutamate burst. Muscimol and glutamate applications are marked by black or green arrows, respectively. **(C)** Original trace of current ramps and resulting voltage responses applied under control conditions (blue trace) and at the peak of a single muscimol response (red trace) before and 600 ms after muscimol burst at higher temporal resolution. **(D)** Statistical analysis of the relative GABAergic rheobase shift (normalized to the rheobase) before and after muscimol and/or glutamate burst. A significant effect of muscimol and/or glutamate on the rheobase (*F*_(2,10)_ = 4.372, *p* = 0.043) and between pre- and post-burst conditions (*F*_(1,11)_ = 31.321, *p* < 0.001) was determined with a repeated-measure ANOVA test. Note that the GABAergic rheobase shift significantly decreased after glutamate, muscimol and the combined muscimol/glutamate burst. **(E)** Original trace of current ramps and resulting voltage responses before and 600 ms after muscimol to estimate the direct effect of the stimulation paradigm on the rheobase under control conditions. **(F)** Statistical analysis of the relative rheobase (normalized to rheobase before burst). Note that the rheobase was significantly increased after bursts of muscimol applications, while burst application of glutamate had no effect. No significant differences (*F*_(2,12)_ = 2.305, *p* = 0.142) between the condition were found with a repeated-measure ANOVA test. Bars represent mean ± SEM. Asterisks on top of values indicate significance from pre-burst value. * and *** indicate significance levels of *p* < 0.05 and *p* < 0.001, respectively; ns, indicate non-significant results.

### Statistics

All data are presented as mean ± SEM. Statistical significance was determined using Student’s *t*-test. Results were designated significant at a level of *p* < 0.05 and significance levels are *p < 0.05, **p < 0.01, and ***p < 0.001. Significance of multiple comparisons was calculated with one-way ANOVA followed by or repeated-measure ANOVA followed by Bonferroni corrected *post hoc* tests (SPSS, IBM).

## Results

### Stable Inhibitory Responses Are Established in Dissociated Neuronal Cultures at DIV13

In a first set of control experiments, we identified the time point at which stable inhibitory GABAergic responses are established in dissociated cell cultures under our experimental conditions. Quantitative rtPCR experiments revealed that the relative levels of KCC2 mRNA in primary neurons increased significantly (*p* = 0.026) from 0.008 ± 0.002 at DIV 7 (*n* = 16 replicates from eight cultures) to 0.016 ± 0.003 (*n* = 15 replicates from eight cultures) at DIV14 (Figure 1A). This upregulation of KCC2 mRNA is also visible by increased immunohistochemical labeling of neurons with KCC2 specific antibodies between DIV7 and DIV14 (Figure 1B), illustrating that also protein levels of KCC2 are upregulated. This KCC2 upregulation was paralleled by the establishment of inhibitory GABAergic responses in dissociated cell cultures, as analyzed in recordings with planar MEAs. The baseline firing frequency of cultured cortical neurons was 0.14 ± 0.03 Hz (*n* = 74 neurons from six cultures) at DIV6–8, which significantly (*p* = 6.2*10^−5^) increased to 0.38 ± 0.03 Hz (*n* = 218 neurons from seven cultures) at DIV13–15 (Figures 1C–E), illustrating the typical developmental up-regulation of network connectivity (Wagenaar et al., 2006; Sun et al., 2010). Inhibition of GABA_A_ receptors by 20 μM gabazine decreased the firing frequency in DIV 6–8 cultures by 69.4 ± 10.6% (*n* = 64 neurons from six cultures), indicating an excitatory effect of GABA_A_ receptors at this developmental stage (Figures 1C,F). In contrast, at DIV13–15 gabazine application increased the firing frequency to 115.8 ± 58.8% (*n* = 271 neurons from seven cultures), suggesting that GABA_A_ receptors mediate an inhibitory effect (Figures 1D,F).

In summary, these results indicate that cortical cultures older than DIV13 show the typical inhibitory GABAergic properties that are characteristic for mature neurons. Therefore, all further experiments were performed at DIV 13–18.

### Repetitive GABA_A_ Receptor Activation Induces Short-Lasting [Cl^−^]_i_ Transients

To limit the variability in the [Cl^−^]_i_, we decided to use whole-cell patch-clamp recordings to stabilize [Cl^−^]_i_ by diffusional exchange with the pipette solution (Pusch and Neher, 1988). In order to demonstrate that this approach will still allow us to detect dynamic [Cl^−^]_i_ changes in the dendrite (Jarolimek et al., 1999), we used a rather high [Cl^−^]_p_ of 20 mM to challenge the Cl^−^ extrusion within the dendritic compartment. To verify that [Cl^−^]_i_ is not clamped in the dendrite, we determined E_Cl_ by a voltage ramp protocol (Figures 2A–C) using focal muscimol application at the soma and at the dendrite ~100 μm apart from the soma (Figure 2D). We measured a mean somatic [Cl^−^]_i_ of 17.6 ± 0.7 mM (*n* = 10 neurons from six cultures) and a significantly (*p* = 0.03) lower dendritic [Cl^−^]_i_ of 15.4 ± 1.3 mM (*n* = 10 neurons from six cultures; Figure 2E), indicating that Cl^−^-extrusion is capable to partially control dendritic [Cl^−^]_i_. Inhibition of Cl^−^-extrusion with the KCC2 antagonist VU 0463271 (10 μM; Sivakumaran et al., 2015) induced an increase in dendritic [Cl^−^]_i_ (Figure 2F). The dendritic [Cl^−^]_i_ significantly (*p* = 1.6*10^−5^) increased from 15.4 ± 1.3 mM (*n* = 10 neurons from six cultures) to 25.8 ± 1.3 mM (*n* = 12 neurons from five cultures) after VU 0463271 application (Figure 2G). These experiments indicate a substantial [Cl^−^]_i_ gradient in the dendrite that was maintained by KCC2 activity (Jarolimek et al., 1999).

All further experiments are performed under whole-cell condition using a [Cl^−^]_p_ of 10 mM, to match the estimated [Cl^−^]_i_ of mature neurons. Under these conditions, the dendritic [Cl^−^]_i_ is maintained at 7.0 ± 0.4 mM (*n* = 54 neurons from 46 cultures). These values increased to 15.0 ± 1.2 mM (*n* = 8 neurons from five cultures) after treatment with 10 μM VU 0463271 (Figure 3A), again indicating the important role of KCC2 for dendritic [Cl^−^]_i_ homeostasis. To reveal the kinetics of KCC2-dependent [Cl^−^]_i_ regulation in the dendritic compartment, we repeatedly induced a [Cl^−^]_i_ shift by burst application (10 × 10 ms at 10 Hz) of 200 μM muscimol at a slightly depolarized V_H_ of −40 mV and determined E_Cl_ at different time points after the muscimol burst application (Figure 3B). The muscimol burst application induced a [Cl^−^]_i_ increase from 6.5 ± 1.1 mM to 15.7 ± 1.0 mM (*n* = 10 neurons from eight cultures; Figure 3C), as determined at 500 ms after the burst. This [Cl^−^]_i_ increase was regulated back to baseline levels of 6.2 ± 0.8 mM within several seconds (Figure 3D). The slope of [Cl^−^]_i_ backregulation amounted to −9.6 mM/s. In the presence of 10 μM VU 0463271 the backregulation after muscimol burst application was slowed down to −1.0 mM/s and reached steady-state values at 15.9 ± 1.5 mM (*n* = 6 neurons from five cultures; Figure 3D), demonstrating the essential role of KCC2 for efficient [Cl^−^]_i_ homeostasis within the dendritic compartment.

In summary, these experiments show that dendritic muscimol bursts induce short-lasting [Cl^−^]_i_ transients even under whole-cell conditions and that KCC2 maintains the resting [Cl^−^]_i_ and determines the fast temporal properties of [Cl^−^]_i_ homeostasis.

### GABA_A_ Receptor-Mediated [Cl^−^]_i_ Transients Are Voltage-Dependent

Since we attempted to use moderate stimulation conditions that are close to physiological levels of GABAergic activity, we limited the muscimol pulses by a fast application system with an efficient local removal. With this system the focal application of muscimol (100 μM, 10 ms) induced a current of 65.4 ± 9.2 pA (*n* = 13 neurons from six cultures), which is only about 2× larger than spontaneous GABAergic IPSCs (31.2 ± 0.3 pA, *n* = 5157 events from four neurons; Figure 4A). In addition, the local removal system limited the spatial dimension of the GABAergic stimulation to <40 μM (Figure 4B), indicating that only a fraction of the dendrite was stimulated. To study the question, whether coincident depolarization by glutamate receptors can in principle affect ionic plasticity upon GABA_A_ receptor activation, we first investigated how E_m_ during muscimol burst application (10 × 10 ms pulses of 100–200 μM muscimol at 10 Hz), influences muscimol-induced [Cl^−^]_i_ transients in dendrites (Figure 4C). These experiments demonstrated a significant [(*F*_(4,32)_ = 39.38), *p* < 0.001, repeated-measure ANOVA] linear dependence between the observed [Cl^−^]_i_ shifts (Δ[Cl^−^]_i_) and the membrane potential during GABA_A_ receptor activation (3.8 ± 1 mM at V_H_ of −60 mV; 7 ± 1.3 mM at −50 mV; 10.7 ± 2 mM at −40 mV; 14.2 ± 2.1 mM at −30 mV; 18 ± 2.7 mM at −20 mV; *n* = 9 neurons; Figure 4D). This result replicates previous observations (Thompson and Gähwiler, 1989; Backus et al., 1998; Kuner and Augustine, 2000) and indicates that GABA_A_ receptor-mediated [Cl^−^]_i_ transients can act as coincidence detectors for synchronous depolarizations.

### Coactivation of Glutamate Receptors Augments GABA_A_ Receptor-Mediated [Cl^−^]_i_ Transients

To directly address our central question whether a glutamatergic coactivation can augment GABA_A_ receptor-mediated [Cl^−^]_i_ transients, we next applied glutamate in a burst-like pattern (10 × 20 ms pulses of 200 μM glutamate at 10 Hz) at identical time points to the muscimol burst application. These burst-like applications of glutamate were performed *via* a pipette located close to the muscimol pipette at the dendrite under current-clamp conditions to allow the cell membrane to integrate GABAergic and glutamatergic postsynaptic potentials. The amplitude of focal glutamate pulses (37.8 ± 9.5 pA, *n* = 11 neurons from six cultures) was comparable to the amplitude of spontaneous glutamatergic EPSCs (39.7 ± 0.5 pA, *n* = 5,273 events from four neurons). Subsequently, [Cl^−^]_i_ was determined from a muscimol application under voltage-clamp conditions using a step protocol (Figure 5A).

These experiments demonstrated that a burst of 10 glutamate applications induced a mean depolarization of 7 ± 1.4 mV (*n* = 27 neurons), whereas burst-like application of 10 muscimol pulses induced a mean hyperpolarization of −3.7 ± 0.2 mV (*n* = 27 neurons). When both substances were applied simultaneously, E_m_ remained mostly unaffected (−0.8 ± 0.7 mV, *n* = 27; Figure 5B), indicating that glutamate co-application substantially affects the driving force on Cl^−^ ions (DF_Cl_). Subsequent [Cl^−^]_i_ measurements (Figure 5A) revealed that a burst of 10 glutamate pulses, as expected, did not affect [Cl^−^]_i_ (Δ[Cl^−^]_*i*_ = 0.1 ± 0.2 mM, *n* = 27 neurons). Application of a burst of 10 muscimol pulses significantly (*p* = 0.025) increased [Cl^−^]_i_ by 3.1 ± 0.4 mM (*n* = 27 neurons). Synchronous application of muscimol and glutamate led to a [Cl^−^]_i_ increase of 4.55 ± 0.53 mM (*n* = 27 neurons; *p* = 4.8*10^−4^; Figure 5C), which is significantly (*p* = 0.042) higher than the [Cl^−^]_i_ transient observed upon only muscimol pulses.

In summary, these results indicate that brief trains of GABAergic inputs induce substantial changes in [Cl^−^]_i_ and that these activity-dependent [Cl^−^]_i_ transients are augmented by glutamate coapplication, suggesting that activity-dependent [Cl^−^]_i_ changes can act as sort of coincidence detector for glutamatergic and GABAergic synaptic inputs.

### GABA_A_ Receptor-Mediated [Cl^−^]_i_ Transients Cause a Reduction of GABAergic Inhibitory Capacity

Finally, we investigated if this [Cl^−^]_i_ increase results in a functionally relevant reduction of the inhibitory capacity mediated by GABA_A_ receptors. In these experiments, we quantified the inhibitory capacity of GABA_A_ receptors as GABAergic rheobase shift. For that purpose, we determined the rheobase (I_RB_), i.e., the threshold current to evoke an AP, from a ramp-like current protocol. An inhibitory effect will require larger currents to reach AP threshold, thus increasing I_RB_. We determined I_RB_ under control conditions (I_RB_^Ctrl^) and in the presence of muscimol (I_RB_^GABA^), which allows us to quantify the inhibitory capacity provided by GABA_A_ receptors (Figure 6A). Upon application of a muscimol pulse, I_RB_ increased significantly (*p* = 2.1*10^−5^) from 364.7 ± 25.5 pA to 721.9 ± 67.2 pA (*n* = 12 neurons) corresponding to a GABAergic rheobase shift (I_RB_^GABA^ − I_RB_^Ctrl^) by 197.5 ± 12.8%. This finding indicates that activation of GABA receptors promotes a substantial inhibitory effect on the excitability of neurons. Next, we determined whether a burst-application of 10 muscimol pulses affects GABAergic rheobase shift, i.e., the inhibitory effect of GABA (Figures 6B,C). Burst application of 10 muscimol pulses induced a significant (*p* = 0.033) reduction in the GABAergic rheobase shift from 197.5 ± 12.8 to 160.9 ± 8.7% (*n* = 12 neurons; Figure 6D). When the burst-like application of muscimol was paired with coincident glutamate applications, the GABAergic rheobase shift decreased significantly (*p* = 5.4*10^−4^) from 201.9 ± 14.5% to 160.8 ± 12.3% (*n* = 12 neurons; Figure 6D), which is not significantly different from the application of muscimol alone (*p* = 0.98). Determination of the rheobase after a burst of 10 glutamate applications demonstrated significantly smaller (*p* = 0.037) decrease of the GABAergic rheobase shift from 197.6 ± 11.2% to 180.7 ± 10.8% (*n* = 12 neurons; *p* = 3.3*10^−4^, Figure 6D).

In order to exclude that a direct effect of either membrane potential alterations or the GABA_A_ receptor-dependent [Cl^−^]_i_ changes on I_RB_ (Sørensen et al., 2017) contributed to the observed reduction in the GABAergic rheobase shift, we also analyzed I_RB_^Ctrl^ before and after the burst-like application protocol. We found that I_RB_^Ctrl^ was significantly (*p* = 0.0119) increased by 8.1 ± 2.3% (*n* = 7 neurons) after burst-like application of muscimol, as compared to values before burst application (Figures 6E,F). The combined burst application of muscimol and glutamate induced a significant (*p* = 0.0128) increase in the I_RB_^Ctrl^ by 6.8 ± 1.4% (*n* = 7 neurons), which is not significantly (*p* = 0.55) different from burst-like muscimol application. Burst-like application of only glutamate had no significant (*p* = 0.35) effect on I_RB_^Ctrl^ (102.6 ± 1.8%; *n* = 7 neurons). These experiments show that burst-like application of muscimol results in a small increase in the rheobase, which can have only a negligible contribution to the observed GABAergic rheobase shift under these conditions.

In summary, these results indicate that brief trains of GABAergic inputs induce a substantial reduction in the GABAergic inhibitory capacity. However, an additional effect of coincident glutamate applications could not be observed.

## Discussion

We used whole-cell patch-clamp recordings of cortical neurons in primary culture to analyze the effect of repetitive GABA_A_ receptor activation on [Cl^−^]_i_ and the inhibitory capacity of GABA_A_ receptors, particularly considering a modulation of these effects by coincident glutamate receptor activation. These experiments revealed, that: (i) considerable [Cl^−^]_i_ shifts could be induced by application of 10 GABAergic pulses under whole-cell conditions; (ii) that this GABA-induced [Cl^−^]_i_ transients were augmented at depolarized membrane potentials; (iii) that synchronous glutamate-applications augment the GABA-induced [Cl^−^]_i_ transients; (iv) that the GABA-induced [Cl^−^]_i_ transients decreased the inhibitory potential of GABA, but that (v) the synchronous coapplication of glutamate pulses had no additional effect on the attenuation of the inhibitory potential of GABA. In summary, these results indicate that moderate GABAergic stimulation induces transient and functionally relevant [Cl^−^]_i_ changes, which can be enhanced by coincident glutamatergic input.

We performed the experiments in neuronal cultures that were cultivated for at least 13 days, as neurons in these cultures display increased KCC2 expression and stable inhibitory GABAergic effects. The development of GABAergic inhibition during maturation *in vitro* is comparable to previous observations of [Cl^−^]_i_ and KCC2 expression in dissociated neuronal cultures (Kuner and Augustine, 2000; Ganguly et al., 2001; Titz et al., 2003). Although dissociated neuronal cultures differ in their synaptic connectivity from those *in vivo*, we decided to use this reduced preparation because it allows us to perform a spatially concise and selective activation of GABA_A_ and/or glutamate receptors at a defined position in the dendrite. By using a local suction system to limit the spatial and temporal extent of the focal pressure application and by using rather short muscimol and or glutamate pulses, we were able to apply moderate GABAergic and glutamatergic stimuli. However, while the amplitudes of currents induced by focal muscimol or glutamate application are maximally two-fold larger than the average amplitudes of spontaneous synaptic currents, it must be emphasized that the focal pressure application does not represent physiological synapse-like stimulation. On one hand, the duration of the focal neurotransmitter applications is considerably longer than spontaneous PSCs and on the other hand, the spatial extent of the applied neurotransmitter spans about 40 μsm, thereby also activating extrasynaptic receptors.

Although the [Cl^−^]_i_ is in general tightly controlled by diffusional exchange with the pipette solution (Pusch and Neher, 1988), our whole-cell patch-clamp experiments revealed that the dendritic [Cl^−^]_i_ was substantially different from the somatic [Cl^−^]_i_, as had been demonstrated previously (Jarolimek et al., 1999; Moore et al., 2018). While the somatic [Cl^−^]_i_ was close to the [Cl^−^]_p_ for [Cl^−^]_p_ values of 10 mM or 20 mM, the dendritic [Cl^−^]_i_ levels were significantly smaller under both conditions. The observation that this somato-dendritic [Cl^−^]_i_ gradient was abolished in the presence of VU0463271 indicates that it depends on KCC2 activity (Moore et al., 2018). In addition, substantial [Cl^−^]_i_ transients could be induced by repetitive muscimol pulses. These observations strongly suggest that the dendritic [Cl^−^]_i_ was only partially affected by the [Cl^−^]_p_. Therefore, we decided to perform all further experiments under whole-cell conditions instead of gramicidin-perforated patch conditions (Ebihara et al., 1995; Kyrozis and Reichling, 1995), as the whole-cell configuration allows more precise electrical control and also allows us to perform the experiments at more defined initial [Cl^−^]_i_. Although such whole-cell experiments may underestimate the real GABA-induced dendritic [Cl^−^]_i_ transients, our observation that the fast recovery of GABA-induced [Cl^−^]_i_ transients was abolished by the KCC2 inhibitor VU0463271 strongly suggests that the down-regulation of dendritic [Cl^−^]_i_ was dominated by transmembrane, KCC2-dependent Cl^−^-transport. Diffusional exchange with the soma (Lombardi et al., 2019), and thus a putative contribution of somatic [Cl^−^]_i_ clamped by the [Cl^−^]_p_, has most probably a negligible contribution to dendritic [Cl^−^]_i_ dynamics.

One major finding of this study is the observation that a moderate GABAergic stimulation can induce substantial [Cl^−^]_i_ transients. In accordance with other studies, we observed that the GABA_A_ receptor-mediated [Cl^−^]_i_ transients were altered if E_m_ was set to depolarized and/or hyperpolarized values under voltage-clamp conditions (Backus et al., 1998; Kuner and Augustine, 2000; Raimondo et al., 2012a). However, the muscimol-induced [Cl^−^]_i_ transients are definitely overestimated under voltage-clamp conditions, because DF_Cl_ was artificially stabilized by preventing voltage changes. But in the present study, we also applied the repetitive muscimol pulses under current-clamp conditions, when GABAergic currents shifted E_m_ towards E_GABA_ and thus reduce DF_Cl_. These experiments revealed that even under these more physiological conditions, substantial [Cl^−^]_i_ transients are induced by activation of GABA_A_ receptors. In the absence of voltage-clamp conditions several factors prevent that DF_Cl_ will fade. Most importantly, the GABAergic hyperpolarization does not reach E_Cl_ due to the persisting HCO_3_^−^ efflux *via* activated GABA_A_ receptors (Grover et al., 1993; Rivera et al., 2005; Wright et al., 2011). In addition, the input conductances will stabilize E_m_ and thus limit the GABAergic depolarization (Lombardi et al., 2019).

The second major finding of the present study is the observation that a coincident co-application of glutamate enhanced the muscimol-induced [Cl^−^]_i_ transients. To our knowledge a direct effect of synchronous glutamatergic depolarizations on GABA-induced [Cl^−^]_i_ transients has not been shown before, although it has been reported that the simultaneous activation of tonic GABAergic currents and glutamatergic currents by long-lasting glutamate applications induces massive [Cl^−^]_i_ changes (Deeb et al., 2011). Although from theoretical consideration each depolarizing current will enhance DF_Cl_ and thus the Cl^−^ fluxes through GABA_A_ receptors (Farrant and Kaila, 2007; Kaila et al., 2014a), the present results are the first report that even moderate levels of glutamatergic stimulation, which were almost comparable to physiologically relevant levels of glutamatergic synaptic activity, are sufficient to enhance GABAergic [Cl^−^]_i_ transients. *via* this mechanism, the [Cl^−^]_i_ may serve as a kind of coincidence detector for glutamatergic and GABAergic inputs. If GABAergic and glutamatergic synapses are simultaneously activated, the GABA_A_ receptor-dependent [Cl^−^]_i_ increase is augmented and thus a putative attenuation of the inhibitory capacity of GABA_A_ receptors would be stronger. This coincident glutamatergic stimulation is in line with other mechanisms that promote a depolarized E_m_ during GABAergic stimuli, like HCO_3_^−^-currents or activity-dependent [K^+^]_e_ transients (Kaila et al., 1997; Rivera et al., 2005; Wright et al., 2011), one additional factor enhancing activity-dependent shifts in GABAergic functions during moderate and massive synaptic activation.

The reduced inhibitory capacity induced by this activity-dependent [Cl^−^]_i_ increase may be an important element of short term plasticity (Jedlicka and Backus, 2006; Wright et al., 2011). Such a temporally limited and stimulus-dependent reduction of inhibition can e.g., attenuate feedforward and/or lateral inhibition and contribute to the gating of relevant information. In addition, this kind of ionic plasticity can also balance the short-term depression of glutamate release at synapses with high release probability (Gil et al., 1999). Thereby the observed influence of glutamatergic activity on ionic plasticity may serve to maintain excitation/inhibition ratio. Finally, the elevated [Cl^−^]_i_ upon ionic plasticity will enable larger glutamatergic depolarizations under co-activation of GABA and glutamate synapses, which may also facilitate the induction of long-term potentiation (Grover and Yan, 1999; Meredith et al., 2003; Ferando et al., 2016).

While the strong or pathophysiological stimuli used in most other studies result in massive changes in the [Cl^−^]_i_ (Ballanyi and Grafe, 1985; Isomura et al., 2003; Raimondo et al., 2015), the moderate GABAergic stimulation in our approach results in smaller and faster [Cl^−^]_i_ transients. Recovery of [Cl^−^]_i_ upon massive glutamatergic stimulation was still incomplete after 3 min (Moore et al., 2018) and the recovery after optogenetic [Cl^−^]_i_ loading showed requires ~30 s (Raimondo et al., 2012a). In contrast, the present study demonstrated that the [Cl^−^]_i_ transients evoked by moderate GABAergic stimulation recovers within ~5 s. This observation is in accordance with previous studies that reported that the [Cl^−^]_i_ transients upon a slightly stronger stimulus under voltage-clamp conditions also recovered within 15 s (Kuner and Augustine, 2000). We assume, that the faster recovery in our experiments reflects smaller [Cl^−^]_i_ transients with a restricted spatial extent. Another important observation of our study in this respect is the fact that the [Cl^−^]_i_ recovery of the activity-dependent [Cl^−^]_i_ transients is impaired in the presence of the KCC2 inhibitor VU0463271. This result implies that KCC2-mediated transmembrane transport constitutes the major factor in [Cl^−^]_i_ recovery, while a diffusion towards the soma is negligible. Thereby the fast KCC2-dependent recovery of [Cl^−^]_i_ determines the temporal properties of ionic plasticity. From the time course and functional consequences of the activity-dependent [Cl^−^]_i_ transients after moderate GABAergic stimuli, it is reasonable to consider that they contribute to short-term plasticity in the adult GABAergic system (Jedlicka and Backus, 2006; Wright et al., 2011).

The inhibition induced by GABA_A_ receptors is mediated *via* membrane hyperpolarization and membrane shunting (Farrant and Kaila, 2007), with the membrane hyperpolarization depending on DF_Cl_, i.e., of the difference between E_m_ and E_Cl_. Therefore, the [Cl^−^]_i_ increase after the repetitive stimulus will reduce the membrane hyperpolarization and thereby decrease the inhibitory capacity of GABAergic responses. Accordingly, we observed that the GABAergic rheobase shift, as a measure for the inhibitory capacity, was significantly attenuated by repetitive GABAergic stimulation. However, in all experiments GABA remained inhibitory, reflecting the fact that the shunting effect ensures that even depolarizing GABAergic responses mediate inhibition, as long as E_GABA_ is negative to the AP threshold (Kolbaev et al., 2011a). It has been recently described that [Cl^−^]_i_ interferes with the excitability not only by its influence on E_GABA_, but that the [Cl^−^]_i_ directly affects the AP threshold (Sørensen et al., 2017). While these experiments indicate that the increased [Cl^−^]_i_ induced by the activation of GABA_A_ receptors should lead to a more hyperpolarized AP threshold and thus enhance excitability (Sørensen et al., 2017), we observed a higher rheobase, i.e., a lower excitability, after repetitive GABAergic stimulation. Probably the small [Cl^−^]_i_ changes observed in our experiments are insufficient to affect AP threshold (Sørensen et al., 2017).

However, while this significantly larger [Cl^−^]_i_ transient upon coincident glutamate/GABA receptor activation should in principle lead to a larger reduction in the inhibitory capacity of GABA_A_ receptors, our experiments failed to find a significant reduction in the GABAergic rheobase shift after this stimulation paradigm. We propose that two electrophysiological properties may underlie this observation. First, the increased [Cl^−^]_i_ upon the coincident activation transfers in a non-linear fashion to only a slightly smaller membrane hyperpolarization (according to the Goldman-Hodgin-Katz equation; Farrant and Kaila, 2007). And second, the inhibitory effect of GABA was mediated by hyperpolarizing and by shunting inhibition (Farrant and Nusser, 2005; Mortensen et al., 2012). Thus the smaller hyperpolarizing inhibition upon a [Cl^−^]_i_ increase was probably masked by the remaining shunting inhibition and may thus limit the effects of moderate [Cl^−^]_i_ changes on GABAergic inhibition.

One additionally relevant information from our experiment is the fact that KCC2 plays an essential role for fast recovery of [Cl^−^]_i_ transients and therefore is the major constituent that limits ionic plasticity. This observation suggests that the amount of inhibition under frequent GABAergic synaptic inputs as well as the short-term depression of GABAergic inhibition upon short bursts of GABAergic inputs is controlled by the activity of KCC2. Therefore it is reasonable to suggest that even small alterations in the capacity of KCC2-mediated Cl^−^-extrusion can impact information processing *via* prolonged and enhanced activity-dependent [Cl^−^]_i_ transients within the dendritic compartment without leading to overt systematic [Cl^−^]_i_ changes, as has been demonstrated *in silico* (Doyon et al., 2016). Such less-controlled activity-dependent [Cl^−^]_i_ changes may also underlie the generation of seizure-like activity in patients with KCC2 mutations (Kahle et al., 2014b; Puskarjov et al., 2014b).

## Data Availability Statement

The datasets generated for this study are available on request to the corresponding author.

## Ethics Statement

The animal study was reviewed and approved by Landesuntersuchungsanstalt RLP, Koblenz, Germany.

## Author Contributions

WK and HL designed this study. LH performed the patch-clamp recordings. CA and AS performed the MEA recordings and the rtPCR experiments. LH, AS and WK analyzed the data. LH, AS, HL and WK wrote the manuscript.

## Conflict of Interest

The authors declare that the research was conducted in the absence of any commercial or financial relationships that could be construed as a potential conflict of interest.
